# Degenerative Cervical Myelopathy Awareness in Primary Care: UK National Cross-Sectional Survey of General Practitioners

**DOI:** 10.2196/58802

**Published:** 2024-08-19

**Authors:** Remi M Rufus-Toye, Amir Rafati Fard, Oliver D Mowforth, Luke V McCarron, Kayen Chan, Yuri Hirayama, Emma K Smith, Munashe Veremu, Benjamin M Davies, Jamie F M Brannigan

**Affiliations:** 1 Division of Neurosurgery Department of Clinical Neurosciences Addenbrooke’s Hospital, University of Cambridge Cambridge United Kingdom; 2 University Hospitals Birmingham NHS Foundation Trust Birmingham United Kingdom; 3 School of General Practice NHS Health Education East of England Cambirdgeshire United Kingdom; 4 Nuffield Department of Clinical Neurosciences University of Oxford Oxford United Kingdom

**Keywords:** cervical spine, degeneration, general practice, myelopathy, neurology, neurosurgery, medical education, cervical myelopathy, primary care, misdiagnosis

## Abstract

**Background:**

Degenerative cervical myelopathy (DCM) is a progressive neurological condition, characterized by spinal cord injury secondary to degenerative changes in the spine. Misdiagnosis in primary care forms part of a complex picture leading to an average diagnostic delay of 2 years. This leads to potentially preventable and permanent disability. A lack of awareness secondary to deficits in postgraduate education may contribute to these delays.

**Objective:**

This study aims to assess the awareness of DCM in the setting of general practice.

**Methods:**

General practitioners completed a quantitative web-based cross-sectional questionnaire. The 17-item questionnaire captured data regarding demographics, subjective awareness, and objective knowledge. The questionnaire was disseminated via professional networks, including via practice managers and senior practice partners. Incentivization was provided via a bespoke DCM fact sheet for those that completed the survey.

**Results:**

A total of 54 general practitioners representing all 4 UK nations responded to the survey. General practitioners most commonly self-assessed that they had “limited awareness” of DCM (n=24, 51%). General practitioners felt most commonly “moderately able” to recognize a case of DCM (n=21, 46%). In total, 13% (n=6) of respondents reported that they would not be at all able to recognize a patient with DCM. Respondents most commonly reported that they were “moderately confident” in their ability to triage a patient with DCM (n=19, 41%). A quarter of respondents reported no prior introduction to DCM throughout their medical training (n=13, 25%). The mean score for knowledge-based questions was 42.6% (SD 3.96%) with the lowest performance observed in patient demographic and clinical recognition items.

**Conclusions:**

General practitioners lack confidence in the recognition and management of DCM. These findings are consistent with the diagnostic delays previously described in the literature at the primary care level. Further work to develop and implement educational interventions to general practitioner practices is a crucial step to improving patient outcomes in DCM.

## Introduction

Degenerative cervical myelopathy (DCM) is a common progressive neurological condition, characterized by compression of the cervical spinal cord secondary to degenerative changes in the spine [1-4]. Prevalence in those over the age of 40 years is estimated to be 5%, making it the most common cause of spinal cord pathology worldwide [1,2,5,6]. Disease progression has profound impacts upon the patients’ quality of life [7-10], resulting in pain, functional neurological decline, and disability.

The nature of pathology lends itself to favorable outcomes with early diagnosis. While existing damage is irreversible, surgery has been demonstrated to halt progression [11,12]. Despite the benefits of surgical intervention, an average diagnostic delay of 2 years has been observed, typically requiring 5 clinic appointments. Misdiagnosis in primary care plays a part in the complex picture leading to delays in the patient pathway [13,14].

Misdiagnosis likely arises due to a lack of awareness, driven by inconsistent terminology [15], similarity with other presentations, and low levels of research activity. Most significantly, recent studies have reported deficient teaching and representation of DCM in medical education curricula [16]. It may be speculated that poor education at medical schools translates to a lack of awareness of DCM among primary care physicians.

To our knowledge, no study has yet characterized the awareness of DCM in primary care. The objective of this study was therefore to assess awareness of DCM in primary care, specifically among general practitioners in the United Kingdom. The aim is to identify knowledge gaps, which may inform initiatives to improve postgraduate knowledge, ultimately improving efficiency of diagnosis and patient outcomes. Improving DCM education is also an important component of the number one research priority of the AO Spine RECODE-DCM international research priority setting initiative: improving awareness [15]*.*

## Methods

### Study Design

The survey was designed following the CHERRIES (Checklist for Reporting Results of Internet E-Surveys) [17].

### Study Partners

#### Myelopathy.org

Myelopathy.org is a global charity with the primary objective of advancing the health and well-being of individuals affected by DCM. The charity places a strong emphasis on enhancing medical education to effectively combat the prevailing challenges of delayed or overlooked diagnosis. This research is a component of a broader initiative that seeks to assess the level of DCM knowledge and awareness among medical practitioners and create strategies to enhance it.

#### Student Society of Myelopathy.org

The student society of myelopathy.org works to promote the goals of myelopathy.org. It coordinates teaching, student essay prizes, and projects to promote awareness and improve the treatment of DCM. It has designed and initiated this study to help serve these goals.

### Survey Design

It was decided that the most efficient manner to cross-sectionally capture the knowledge and awareness of general practitioners regarding DCM across the United Kingdom was an electronic survey. The survey entailed 2 parts. Part 1 obtained information regarding the demographics of practitioners in addition to their previous experience of the condition. Part 2 provided a multifaceted assessment. First, the knowledge that would be required of a practitioner to successfully recognize a potential case of DCM was probed. Questions therein related to symptomology and signs at presentation. Furthermore, part 2 evaluated a participant’s ability to ensure prompt treatment by capturing knowledge of appropriate means of referral and investigation. Additional focus included questions regarding patient experience of the disease, patient demographics, and practitioner’s subjective assessment of their knowledge.

The survey was compiled using an iterative approach. Questions were developed with input from a general practitioner (EKS) and academic neurosurgeons with a subspecialist interest in DCM (ODM and BMD). Part 1 was refined to the point at which it was felt that enough practitioner related demographic information was obtained to allow analysis of potential confounders. Similarly, part 2 was developed to a point at which it assessed the fundamental knowledge required by a general practitioner to provide appropriate primary care management to a patient with DCM. This resulted in a 17-item questionnaire (Multimedia Appendix 1).

### Survey Piloting

The piloting group consisted of general practitioners who were not involved in study design. It was highlighted during this phase that ambiguity arose in a question regarding referral due to differences across regions regarding musculoskeletal referral pathway (Multimedia Appendix 1). The question was subsequently updated to remove ambiguity before wider dissemination. It was reported that the format was readily accessible on both desktop and laptop. Relevant responses from this pilot group were included in the final analysis where question items were deemed appropriate for the final survey dissemination.

### Survey Administration

The survey was hosted by Momentive on their Survey Monkey platform (Momentive), a commercial web-based survey platform. The platform offers both desktop and smartphone formatting making the survey readily accessible.

The survey contained 17 items, with the final item (being optional) requesting contact details for those who wished to receive a fact sheet regarding DCM for their future practice.

### Dissemination

To achieve widespread dissemination of the survey across the United Kingdom, appropriate professional networks were approached to facilitate electronic distribution of the survey. Practice managers and senior partners, who had no affiliation with the survey, disseminated the survey throughout their networks. While prompted to advertise the survey on one occasion, it was left to the practice managers’ and senior partners’ discretion as to whether follow-up prompts were administered.

Professional networks were engaged in a manner to capture representation of general practitioners across a wide geographical base. Representation of general practitioners was obtained from all 4 nations. Responses were collected from the period of September 2022 to July 2023.

As an incentive to complete the questionnaire, participants were offered a fact sheet containing the key knowledge required at the level of primary care about the condition, on completion of the survey. It was felt that this was an appropriate incentive which avoided compromising the integrity of the survey while also helping to achieve one of the goals of Myelopathy.org in improving awareness and treatment of DCM. This was agreed by the study management group and the research ethics committee.

### Eligibility and Representation

Any general practitioner working within the United Kingdom was eligible to complete the survey. We aimed to achieve representation of all 4 UK nations.

### Ethical Considerations

Ethical approval was granted by the University of Cambridge Psychology Research Ethics Committee (application: PRE.2022.115).

### Consent and Confidentiality

The participant information sheet highlighted the importance, objectives, and voluntary aspects of participation, while being cautious in selecting background information about DCM to avoid biasing responses for the knowledge-based survey questions. To ensure both anonymity and confidentiality, participants created a distinctive identifier using specific details from their mother’s maiden name and smartphone number, enabling the potential linkage of future surveys by the same participant.

### Data Security

The data remained solely on the secure, web-based Survey Monkey platform until the survey closed. Subsequently, the survey data was transferred from the Survey Monkey platform to a password-protected computer, using an Excel (Microsoft Corp) spreadsheet. Access to this data was strictly limited to the immediate research team on an as-needed basis. Once the data analysis was completed, any unnecessary data was permanently deleted. Importantly, no participant-identifiable data was collected or stored.

### Statistical Analysis

Data analysis and visualization were performed using R (version 4.3.1, The R Foundation) and RStudio (version 2023.06.1, RStudio Team).

Fisher exact test was used to assess for associations between respondent characteristics and survey responses. This was used in place of a chi-square statistic due to the small sample size of the data. Where comparisons between respondent characteristics and responses expressed quantitatively as proportions of correct answers were sought, the Kruskal-Wallis ANOVA was employed, as the data were nonparametric. Errors reported are SEM.

## Results

### Summary of Respondent Characteristics

The survey captured the responses of 54 general practitioners, representing all 4 nations of the United Kingdom (England: 30%, n=16; Northern Ireland: 65%, n=35; Scotland: 4%, n=2; and Wales: 2%, n=1). Responses came mostly from experienced general practitioners: 9 (17%) were still in training, 6 (11%) were under 5 years post training, 7 (13%) were 5-10 years post training, 20 (37%) were 11-20 years post training, and 12 (22%) were more than 20 years post training. The full data set can be found in Multimedia Appendix 2.

### Training and Exposure

The stage of training at which respondents were first introduced to DCM varied. Respondents were most commonly introduced to DCM in medical school (n=16, 30%), while 43% (n=23) reported being introduced to DCM during training after medical school (Table 1). A quarter of respondents reported no prior introduction to DCM throughout their medical training (n=13, 25%). Most general practitioners (n=27, 51%) reported at least 1 encounter with suspected DCM per month (Table 2). In total, 49% (n=26) respondents reported encountering 0 patients per month with suspected DCM.

**Table 1:** General practitioner responses to the question “At which stage of your training were you introduced to DCM, also historically known as cervical spondylitic myelopathy?”

Training stage at introduction	Respondents, n (%)
Medical school	16 (30)
Foundation years or house officer	4 (8)
General practitioner trainee	12 (23)
General practitioner post-training	7 (13)
Never	13 (25)
Other	1 (2)

**Table 2:** General practitioner responses to the question “Approximately how many patients with suspected DCM do you encounter, per month, in clinical practice?”

Patients seen per month	Respondents, n (%)
0	26 (49)
1	12 (23)
2	8 (15)
3	3 (6)
4	1 (2)
5	1 (2)
Other	2 (4)

### Subjective Awareness

Survey respondents were asked to subjectively assess their levels of awareness of DCM. The most common response was “limited awareness” (n=24, 51%; Table 3). A total of 3 respondents reported “no awareness” (6%). No respondents reported having an “excellent awareness” of the condition. There was no significant association between the number of years of training of respondents and their subjectively rated awareness of DCM (*P*=.15).

Respondents were also asked to subjectively rate their ability to recognize DCM. The most common response was “moderately able” (n=21, 46%; Table 4). No respondents felt that they were “extremely able”; however, 13% (n=6) reported that they would be “not at all able” to recognize a patient with DCM.

**Table 3:** General practitioner responses to the question “How would you currently rate your awareness of myelopathy/degenerative cervical myelopathy (DCM)?”

Self-assessed awareness	Respondents, n (%)
Excellent awareness	0 (0)
Very good awareness	4 (9)
Average awareness	16 (34)
Limited awareness	24 (51)
No awareness	3 (6)

**Table 4:** General practitioner responses to the question “How do you currently rate your ability to recognize myelopathy/degenerative cervical myelopathy (DCM)?”

Self-assessed ability to recognize DCM^a^	Respondents, n (%)
Extremely able	0 (0)
Very able	2 (4)
Moderately able	21 (46)
Slightly able	17 (37)
Not at all able	6 (13)

^a^DCM: degenerative cervical myelopathy.

Finally, respondents self-assessed their confidence in triaging a patient with DCM. Few respondents felt “very confident” (n=4, 9%) or “extremely confident” (n=1, 2%) in triage (Table 5). Respondents most reported that they were “moderately confident” in their ability to triage a patient with DCM (n=19, 41%). There was no significant association between the number of years of training of respondents and their subjectively rated ability to recognize DCM (*P*=.53).

**Table 5:** General practitioner responses to the question “If you suspect a case of DCM, how confident are you currently at triaging that patient (i.e., knowing where to refer them and how quickly)?”

Self-assessed confidence to triage patients with DCM^a^	Respondents, n (%)
Extremely confident	1 (2)
Very confident	4 (9)
Moderately confident	19 (41)
Slightly confident	14 (30)
Not at all confident	8 (17)

^a^DCM: degenerative cervical myelopathy.

### Objective Awareness

**Figure 1:**
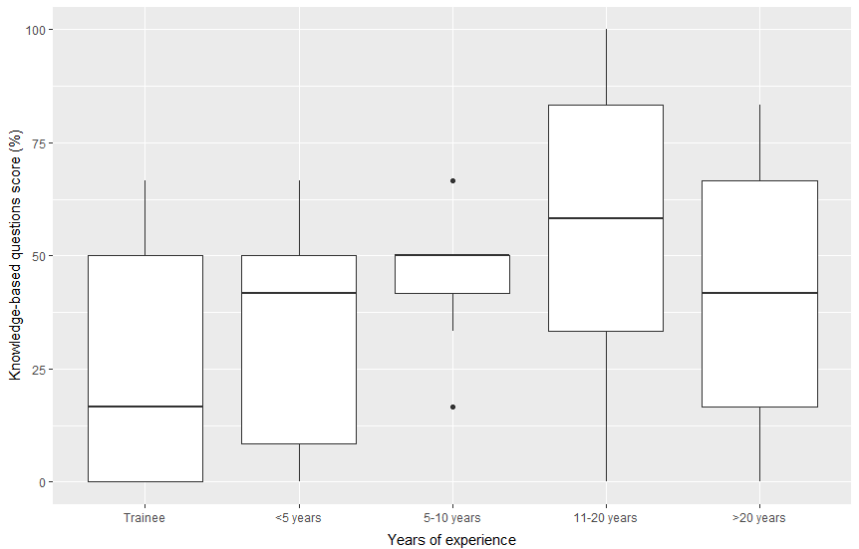
General practitioner performance in knowledge-based questions stratified by years of experience.

When asked 6 objective questions to assess knowledge of DCM, the mean score of respondents was 42.6% (SD 4%). There was no statistically significant correlation between performance and years of training (*χ*^2^_4_=5.6; *P*=.23; Figure 1).

Performance varied by question item and topic area. The lowest performance was observed in question items relating to patient demographics and clinical recognition. A total of 19% (n=9) correctly identified the prevalence of DCM in those aged over 40, while 50% of respondents (n=21) answered the correct clinical sign to differentiate DCM from carpal tunnel syndrome (Multimedia Appendix 2).

In contrast, 78% of respondents (n=36) correctly identified the most important modality of imaging to diagnose DCM. Furthermore, 74% of respondents (n=31) correctly identified that referral to neurosurgery would be the most appropriate form of triage for a patient who has been diagnosed with DCM.

## Discussion

### Principal Findings

One of the major challenges currently facing people with DCM is delayed diagnosis. For a progressive, largely irreversible condition with treatment available that can halt progression, early diagnosis and specialist care is fundamentally important for good patient outcomes [18]. Misdiagnosis is part of a complex picture leading to delayed diagnosis [13]. Our findings suggest that a lack of awareness about DCM and a low ability to recognize the condition may contribute to misdiagnosis in primary care. General practitioners also reported a lack of training on DCM, with a substantial proportion of respondents reporting that they had not encountered DCM at any point in their training. Despite low confidence and awareness about DCM, respondents demonstrated a reasonable level of performance on questions related to disease management.

### General Practitioners Report Difficulties in Recognizing DCM

A key finding from our survey was that general practitioners have low self-assessed ability to recognize a case of DCM. Only a small minority (n=2, 4%) felt they were “very able” or “extremely able” to recognize DCM, with 50% (n=23) feeling either “slightly able” or “not able at all” (Table 4). This was reflected in the results of questionnaire items objectively assessing ability to recognize DCM. When asked to identify features of patient presentation that may differentiate DCM from carpal tunnel syndrome, only 50% (n=21) of respondents responded correctly. These observations could not be explained by respondents’ level of experience. Neither confidence in recognition nor performance in knowledge-based questions were statistically significantly correlated with the number of years of experience as a general practitioner.

Previous work has detailed the pathway to definitive management of DCM, with delay secondary to misdiagnosis in primary care being a factor [13,19]. Of note, the most common diagnosis received was carpal tunnel syndrome [13]. We propose that this delay may be due to a lack of knowledge regarding the clinical presentation of DCM.

Prior gap analysis has demonstrated a deficiency in references to DCM in both educational curricula and study materials [20]. Our results corroborate these findings, with 25% (n=13) of respondents reporting never having been introduced to DCM at any point in their training (Table 1). This large absence of education may be an underlying cause for the difficulties that general practitioners have expressed and demonstrated in this study.

It is of note that our respondents included general practitioner trainees. Incomplete training from respondents introduces the potential to confuse our results. However, of the respondents who reported having never been introduced to DCM, only 1 respondent (8%) was a trainee.

### General Practitioners Report Low Confidence in the Management of DCM

General practitioners did not feel confident in appropriately triaging patients with DCM. A total of 41 general practitioners (89%) felt at best “moderately confident” in triage, while 8 (17%) felt “not confident at all” (Table 5). Despite low confidence, respondents performed comparatively well on questions relating to investigation and management of DCM, where 31 respondents (74%) correctly identified appropriate modes of referral (Multimedia Appendix 2).

This discrepancy between the confidence expressed by general practitioners to correctly triage patients, and their responses to knowledge-based questions may be explained by the cuing bias that single best answer style questions may introduce to applied medical knowledge assessments [21]. However, it is important to highlight that a large proportion of respondents (n=11, 26%) selected an inappropriate mode of referral which would lead to delays in management [19]. Moreover, the lack of DCM training reported by respondents may have left general practitioners feeling poorly equipped to provide appropriate management to patients with DCM.

### General Practitioners Underestimate Prevalence and Misdiagnosis of DCM

Most respondents answered incorrectly to both the prevalence of DCM (n=38, 81%) and the average time of diagnosis (n=30, 64%).

We believe that an underappreciation of the scale of the problem regarding the delayed diagnosis of DCM may disincentivize efforts among the general practitioner community to address the issues identified. Additionally, an underestimate of the base-rate of disease among the general population may negatively affect probabilistic reasoning, leading to underdiagnosis [22,23]. Efforts to increase understanding of these issues are aligned with the number 1 research priority identified by the AO Spine RECODE-DCM initiative—improving awareness [15]*.*

### Deficits in General Practitioner Confidence and Awareness May Reflect Broader Issues in Neurological Education

Diagnostic delay is not unique to DCM. Other neurological conditions, including dementia, amyotrophic lateral sclerosis, and epilepsy face similar problems [24-26]. Although this is partially attributable to the insidious progression that characterizes many neurological conditions, educational shortcomings have been well described in the literature [27,28].

The phenomenon of neurophobia is believed to be another one of the factors contributing to such shortcomings. This describes a disinclination of medical students and junior doctors to tackle the task of understanding neurological disease [29,30]. This may go some way to explain the consistent lack of confidence revealed from the results of our survey, even when related objective questions were answered more accurately.

Reviews of the literature have demonstrated that there is currently limited evidence regarding effective interventions for neurology education [31]. The development of a strong evidence base for effective interventions in neurology education of all levels would be of great value not just to the prognostics of DCM, but to neurological conditions.

### Limitations and Future Work

To our knowledge, this is the first study that attempts to identify levels of confidence and awareness among general practitioners pertaining to DCM. Our survey relied partially on general practitioners’ self-evaluation of their awareness of DCM, potentially introducing response and recall biases. To enhance the robustness of our findings and mitigate the effects of such bias, we incorporated an objective assessment of awareness into our survey.

Despite widespread dissemination with an appropriate incentive, recruitment of general practitioners proved a challenge, resulting in a relatively small sample size. Challenges in recruitment may be partially attributed to unprecedented clinical pressures facing general practitioners in the United Kingdom [32,33]. This limitation restricts the generalizability of our findings to general practitioners across the United Kingdom and may introduce selection bias. Furthermore, while our survey successfully achieved representation from all nations in the United Kingdom, a significant proportion of this was from general practitioners based in Northern Ireland, which further limits the generalizability of our results.

While our study highlights the relatively low awareness of DCM among general practitioners in the United Kingdom, future work should aim to validate our preliminary findings using a larger and more diverse sample of primary care clinicians. Given our difficulty in attaining even UK wide representation, future studies may benefit from focusing on individual UK regions, allowing targeted use of resources in underrepresented areas.

Literature regarding general practitioner awareness of DCM internationally is sparse. However, previous work suggests that lack of awareness exists in other health care systems in the global north [13]. International surveys may offer valuable insights and opportunities for learning from countries with a comparatively higher awareness of DCM.

Our results suggest a discrepancy between general practitioners’ confidence of primary care management of DCM and performance in objective assessments. To gain a deeper understanding of this phenomenon and the wider factors influencing DCM awareness in the primary care setting, it would be beneficial to supplement questionnaire data with qualitative research methods, including interviews and focus groups. These could be used to define the learning needs of the population.

This understanding provides a clear rationale for educational intervention at the primary care level. Such education must provide clarity and simplification to recognition and management of DCM. No diagnostic criteria currently exist for DCM. It is only recently that DCM was defined [34,35]. We plan to develop diagnostic criteria for DCM, following which we would reassess confidence and awareness of DCM. Considering the limitations in generalizability of this study and its cross-sectional nature, validation of such approaches may require focused assessment of general practitioner awareness and confidence both before and after intervention.

Previous work has noted the significant difference between the neurological examination of specialists versus non specialists, with the former often targeting examination to rule out specific differentials [36,37]. A clear reference tool for general practitioners may aid prompt and streamlined referral onto secondary care, reducing subsequent disability [38-40]. This aligns with the number 3 research priority identified by the AO Spine RECODE-DCM initiative—establishing diagnostic criteria for DCM [38].

### Conclusions

DCM is a rising health concern [41,42] for which general practitioners lack confidence in the recognition and management of DCM. This lack of awareness has clear implications for prompt diagnosis and referral onto specialist care. Addressing the education deficits highlighted by this study is an essential step to resolving these issues.

## Appendix

Multimedia Appendix 1 Final survey design.

Multimedia Appendix 2 Full data set.

## Abbreviations

CHERRIES: Checklist for Reporting Results of Internet E-Surveys
DCM: degenerative cervical myelopathy

## Data Availability

The majority of data generated or analyzed during this study are included in this article. The remaining data sets generated during and/or analyzed during this study are available from the corresponding author on reasonable request.
