# Supplementary figures and images for: Degenerative Cervical Myelopathy Awareness in Primary Care: UK National Cross-Sectional Survey of General Practitioners
Source: JMIR Form Res. 2024 Aug 19;8:e58802. doi: 10.2196/58802 (PMC11369528; doi:10.2196/58802)

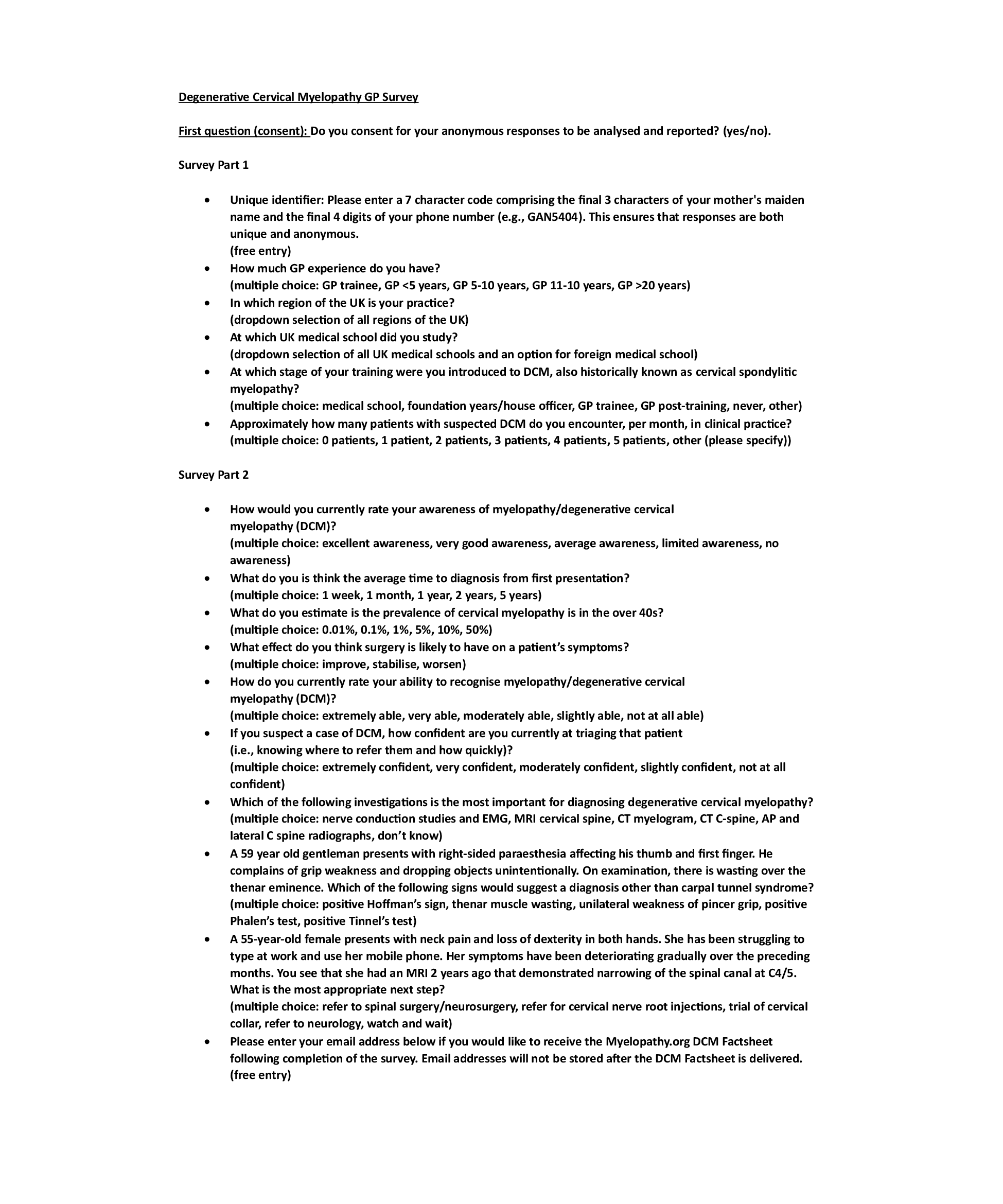

Supplement: Multimedia Appendix 1 [file formative_v8i1e58802_app1.png]
