# Supplementary material for: Degenerative Cervical Myelopathy Awareness in Primary Care: UK National Cross-Sectional Survey of General Practitioners
Source: JMIR Form Res. 2024 Aug 19;8:e58802. doi: 10.2196/58802 (PMC11369528; doi:10.2196/58802)
Supplement: Multimedia Appendix 2 [file formative_v8i1e58802_app2.pdf]

Supplementary Material

How much GP experience do you have?

Answer Choices	Responses	
GP trainee	16.67%	9
GP <5 years	11.11%	6
GP 5-10 years	12.96%	7
GP 11-20 years	37.04%	20
GP >20 years	22.22%	12
	**Answered**	**54**
	**Skipped**	**0**

In which region of the UK is your practice?

Answer Choices	Responses	
Northern Ireland	64.81%	35
Scotland	3.70%	2
Wales	1.85%	1
London	1.85%	1
North East of England	1.85%	1
North West of England	1.85%	1
Yorkshire	0.00%	0
East Midlands	0.00%	0
West Midlands	0.00%	0
South East of England	5.56%	3
East of England	18.52%	10
South West	0.00%	0
	**Answered**	**54**
	**Skipped**	**0**

At which medical school did you study?

Answer Choices	Responses	
University of Aberdeen	1.89%	1
Barts	1.89%	1
Anglia Ruskin	1.89%	1
Aston University	0.00%	0
Univseristy of Birmingham	5.66%	3
Brighton and Sussex	0.00%	0
University of Bristol	0.00%	0
University of Buckingham	1.89%	1
University of Cambridge	1.89%	1
Cardiff University	1.89%	1
University of Dundee	0.00%	0
Edge Hill University	0.00%	0

University of Edinburgh	0.00%	0
University of Exeter	0.00%	0
University of Glasgow	9.43%	5
Hull York	0.00%	0
Imperial College London	3.77%	2
Keele University	0.00%	0
Kent and Medway	0.00%	0
King's College London	1.89%	1
Lancaster University	0.00%	0
University of Leeds	0.00%	0
University of Leicester	0.00%	0
University of Liverpool	0.00%	0
London School of Hygeine and Tropical Medicine	0.00%	0
University of Manchester	1.89%	1
Newcastle University	0.00%	0
Norwich	0.00%	0
University of Nottingham	0.00%	0
University of Oxford	0.00%	0
Plymouth University	0.00%	0
Queen's University Belfast	35.85%	19
University of Sheffield	0.00%	0
University of Southampton	1.89%	1
University of St. Andrews	0.00%	0
St. George's	1.89%	1
University of Sunderland	0.00%	0
Swansea University	1.89%	1
University of Central Lancashire	0.00%	0
University College London	5.66%	3
University of Warwick	0.00%	0
Brunel	0.00%	0
University of Ulster	3.77%	2
University of Chester	0.00%	0
Foreign Medical School	15.09%	8
	**Answered**	**53**
	**Skipped**	**1**

At which stage of your training were you introduced to DCM, also historically known as cervical spondylitic myelopathy?

Answer Choices	Responses	
Medical school	30.19%	16
Foundation years/house officer	7.55%	4
GP trainee	22.64%	12
GP post-training	13.21%	7
Never	24.53%	13
Other	1.89%	1

**Answered**	**53**
**Skipped**	**1**

Approximately how many patients with suspected DCM do you encounter, per month, in clinical practice?

Answer Choices	Responses	
0 patients	49.06%	26
1 patient	22.64%	12
2 patients	15.09%	8
3 patients	5.66%	3
4 patients	1.89%	1
5 patients	1.89%	1
Other (please specify)	3.77%	2
	**Answered**	**53**
	**Skipped**	**1**

How would you currently rate your awareness of myelopathy/degenerative cervical myelopathy (DCM)?

Answer Choices	Responses	
Excellent awareness	0.00%	0
Very good awareness	8.51%	4
Average awareness	34.04%	16
Limited awareness	51.06%	24
No awareness	6.38%	3
	**Answered**	**47**
	**Skipped**	**7**

What do you is think the average time to diagnosis from first presentation?

Answer Choices	Responses	
1 week	2.13%	1
1 month	8.51%	4
1 year	44.68%	21
2 years	36.17%	17
5 years	8.51%	4
	**Answered**	**47**
	**Skipped**	**7**

What do you estimate is the prevalence of cervical myelopathy is in the over 40s?

Answer Choices	Responses	
0.01%	10.64%	5
0.1%	8.51%	4
1%	38.30%	18
5%	19.15%	9
10%	21.28%	10
50%	2.13%	1
	**Answered**	**47**
	**Skipped**	**7**

What effect do you think surgery is likely to have on a patient's symptoms?

Answer Choices	Responses	
Improve	17.02%	8
Stabilise	63.83%	30
Worsen	19.15%	9
	**Answered**	**47**
	**Skipped**	**7**

How do you currently rate your ability to recognise myelopathy/degenerative cervical myelopathy (DCM)?

Answer Choices	Responses	
Extremely able	0.00%	0
Very able	4.35%	2
Moderately able	45.65%	21
Slightly able	36.96%	17
Not at all able	13.04%	6
	**Answered**	**46**
	**Skipped**	**8**

If you suspect a case of DCM, how confident are you currently at triaging that patient (i.e., knowing where to refer them and how quickly)?

Answer Choices	Responses	
Extremely confident	2.17%	1
Very confident	8.70%	4

Moderately confident	41.30%	19
Slightly confident	30.43%	14
Not at all confident	17.39%	8
	**Answered**	**46**
	**Skipped**	**8**

Which of the following investigations is the most important for diagnosing degenerative cervical myelopathy?

Answer Choices	Responses	
Nerve conduction studies and EMG	4.35%	2
MRI cervical spine	78.26%	36
CT myelogram	4.35%	2
CT C-spine	2.17%	1
AP and lateral C-spine radiographs	0.00%	0
Don't know	10.87%	5
	**Answered**	**46**
	**Skipped**	**8**

A 59 year old gentleman presents with right-sided paraesthesias affecting his thumb and first finger. He complains of grip weakness and dropping objects unintentionally. On examination, there is wasting over the thenar eminence. Which of the following signs would suggest a diagnosis other than carpal tunnel syndrome?

Answer Choices	Responses	
Positive Hoffmans sign	50.00%	21
Thenar Muscle Wasting	21.43%	9
Unilateral weakness of pincer grip	19.05%	8
Positive Phalens test	4.76%	2
Positive Tinnel’s test	4.76%	2
	**Answered**	**42**
	**Skipped**	**12**

A 55-year-old female presents with neck pain and loss of dexterity in both hands. She has been struggling to type at work and use her mobile phone. Her symptoms have been deteriorating gradually over the preceding months. You see that she had an MRI 2 years ago that demonstrated narrowing of the spinal canal at C4/5. What is the most appropriate next step?

Answer Choices	Responses	
Refer to spinal surgery/neurosurgery	73.81%	31
Refer for cervical nerve root injections	7.14%	3
Trial of a cervical collar	0.00%	0
Refer to neurology	16.67%	7
Watch and wait	2.38%	1
	**Answered**	**42**
	**Skipped**	**12**
